# Scale-Up and Scale-Out of a Gender-Sensitized Weight Management and Healthy Living Program Delivered to Overweight Men via Professional Sports Clubs: The Wider Implementation of Football Fans in Training (FFIT)

**DOI:** 10.3390/ijerph17020584

**Published:** 2020-01-16

**Authors:** Kate Hunt, Sally Wyke, Christopher Bunn, Craig Donnachie, Nicky Reid, Cindy M. Gray

**Affiliations:** 1 Institute for Social Marketing, University of Stirling, Stirling FK9 4LA, UK; 2 Institute of Health and Wellbeing, University of Glasgow, Glasgow G12 8QQ, UK; sally.wyke@glasgow.ac.uk (S.W.); Christopher.bunn@glasgow.ac.uk (C.B.); Craig.Donnachie@glasgow.ac.uk (C.D.); cindy.gray@glasgow.ac.uk (C.M.G.); 3 Department of Psychology, Curtin University, Bentley, WA 6102, Australia; 4 Scottish Professional League Trust (SPFL-T), Glasgow G42 9DE, UK; NickyR@spfltrust.org.uk

**Keywords:** obesity, men’s health, weight loss interventions, health behavior change, physical activity, context, implementation, scalability and sustainability of interventions, scale-up, scale-out

## Abstract

Increasing prevalence of obesity poses challenges for public health. Men have been under-served by weight management programs, highlighting a need for gender-sensitized programs that can be embedded into routine practice or adapted for new settings/populations, to accelerate the process of implementing programs that are successful and cost-effective under research conditions. To address gaps in examples of how to bridge the research to practice gap, we describe the scale-up and scale-out of Football Fans in Training (FFIT), a weight management and healthy living program in relation to two implementation frameworks. The paper presents: the development, evaluation and scale-up of FFIT, mapped onto the PRACTIS guide; outcomes in scale-up deliveries; and the scale-out of FFIT through programs delivered in other contexts (other countries, professional sports, target groups, public health focus). FFIT has been scaled-up through a single-license franchise model in over 40 UK professional football clubs to 2019 (and 30 more from 2020) and scaled-out into football and other sporting contexts in Australia, Canada, New Zealand, England and other European countries. The successful scale-up and scale-out of FFIT demonstrates that, with attention to cultural constructions of masculinity, public health interventions can appeal to men and support them in sustainable lifestyle change.

## 1. Introduction

### 1.1. Background

Rising levels of physical inactivity, sedentary behavior and consumption of foods that contain high levels of sugar and fat have contributed to rising levels of obesity worldwide [1]. Obesity and inactivity are major risk factors for ill-health and mortality from a wide range of non-communicable diseases, at high cost to individuals, families, communities and society [2]. These trends in obesity, physical activity and eating patterns have been driven by major societal forces such as technological change that has reshaped the working environment and transportation, and the global reach of parts of the food industry that heavily promote processed food high in sugar and fat, sugar-sweetened beverages and alcohol, including through professional sport [3,4]. These forces need to be tackled by policy at a population and organizational level. Nonetheless individual behaviors, themselves rooted in cultural and social practices, contribute to health, wellbeing and longevity, and are amenable to change with culturally-sensitized interventions.

In developed countries, the prevalence of being overweight (body mass index (BMI) > 25 kg/m^2^) is higher amongst men than women throughout adult life, and notably so between the ages of around 20 to 60 years [1], but men have been under-represented in commercial and other weight management programs [5,6,7]. The belief that men do not want to take part in healthy lifestyle programs, in particular group-based programs, has been widespread, yet increasing evidence (including research described here) challenges this viewpoint. In 2009, we developed a weight management and healthy living program, designed to appeal to men in its context, content and style of delivery, and to be delivered through professional football (soccer) clubs [8]. At that time, obesity prevalence in Scotland (69% of men versus 62% of women ‘overweight’ or ‘obese’ [9]), where the program was first delivered, was amongst the highest in Europe.

The Football Fans in Training (FFIT) program has proved to be very successful [10,11,12]. Before going on to describe the development [13], evaluation and subsequent scale-up and scale-out of FFIT to date in relation to two frameworks which were designed to explain and guide widespread implementation, we first briefly describe the problems of implementing evidence-based interventions.

### 1.2. Implementation of Evidence-Based Interventions

The long-term sustainability of effective evidence-based public health interventions has concerned public health researchers for some time (see e.g., [14,15,16,17,18,19]), with clear recognition that “there remains a large research-to-practice gap” [16] (p. 8 of 12). Green et al., for example, depict the research-to practice gap as a ‘leaky pipeline’ and a ’17-year odyssey’ even when research is eventually integrated into practice (see Figure 1 in [20]). They suggested that more practice-based evidence is needed to address this gap rather than research conducted in highly controlled circumstances.

However, addressing the research to practice gap can be challenging. Hailemarin et al. noted one such challenge: “ensuring. transferability to community settings or community-based organizations, while also maintaining fidelity” [16] (p. 8 of 12). Bauer et al. highlighted the trade-offs researchers need to make in the context of finite research funding between “investing in more conservative projects with predictable results versus more innovative research, including projects involving more real-world samples that could result in greater public health impact” [15] (pp. 1–2 of 12). Peters et al. highlighted eight “implementation outcomes” that can guide assessment of the success of implementation or insights into the impacts of an intervention on health outcomes. These are: acceptability (to stakeholders), adoption, appropriateness (perceived fit in a particular setting or for a particular target group), feasibility, implementation cost, coverage and sustainability (the extent to which an intervention is maintained or institutionalized in a given setting) [21]. There has also been increasing recognition of the importance of context in the development, evaluation and translation of public health interventions [22].

A recent review identified eight different frameworks to guide scaling of health interventions [23]. However, as it was published in 2015, this could not include Koorts et al.’s (2019) practical guide for researchers wishing to increase the likelihood of successful implementation and scale-up of physical activity interventions in practice (PRACTIS guide) [17]. The PRACTIS (PRACTical planning for Implementation and Scale-up) guide defines implementation as “the use of strategies to adopt and integrate evidence-based health interventions and change practice patterns within specific settings” (p. 1 of 11) and suggests researchers can achieve successful implementation and scale-up of interventions through four steps: Step 1—Characterize parameters of the implementation setting using the five ‘Ps’: Place (what settings/organizations will be involved/required); People (who/how many people the intervention will reach and which individuals will be involved/required for effective implementation); Process (how the intervention or implementation process will occur); Provisions (what resources may be necessary to achieve this) and Principles (implementation process (e.g., building capacity for implementation) that will be scaled-up); Step 2—Identify and engage key stakeholders across multiple levels within the delivery system(s); Step 3—Identify contextual barriers and facilitators to implementation; and Step 4—Address potential barriers to effective implementation [17] (p. 3 of 11).

In this paper, we also draw on Aarons et al.’s distinction between ‘scaling up’ and ‘scaling out’ [14]. They defined ‘scaling up’ as “the deliberate effort to broaden the delivery of an Evidence Based Intervention (EBI) with the intention of reaching larger numbers of a target audience” (p. 3). In scaling-up, an EBI “designed for one setting … is expanded to other health delivery units within the same or very similar settings under which it has been tested” (p. 2). In considering ways of scaling-up, we consider the distinction highlighted by Hailemariam et al. between sustainability (“the extent to which an evidence-based intervention can deliver its intended benefit over an extended period of time”) and sustainment (“creating and supporting the structures and processes that will allow an implemented innovation to be maintained in a system or organization”) [16] (p. 2 of 12)) to be important.

In contrast to scaling-up, Aarons et al. define ‘scaling-out’ as “the deliberate use of strategies to implement, test, improve and sustain EBIs as they are delivered in novel circumstances *distinct from, but closely related to*, previous implementations” (p. 2, emphasis added); these novel circumstances may entail delivery to “*new populations* and/or through *new delivery systems that differ from those in effectiveness trials*” (p. 3, emphasis in the original). Aarons et al. propose three types of scaling-out: type I involves “targeting the same population as previously tested, but through a different delivery system”; type II involves “targeting a different population than previously tested, but through the same delivery system”; and type III involves “targeting a different population, through a different delivery system, as compared to the original EBI trial” (p. 3). Within all types of scale-out, they argue, three conditions should be met: “First, even as the EBI is adapted to new settings and populations, it still must retain its core elements. Second, the underlying mechanism of action regarding how core elements affect health outcomes should remain the same. Third, there must be sufficient organizational or system support to deliver the intervention as intended to sufficient numbers of the target population” [14] (p. 4). We describe the application of this approach to frame the ‘scaling-out’ of FFIT to different countries, different professional sports settings and different population groups.

### 1.3. Aim of the Paper

The aim of this paper is to describe: (a) the development, evaluation and scale-up of FFIT, retrospectively mapped onto the PRACTIS guide; (b) the outcomes of the scale-up of FFIT; and c) the scale-out of FFIT through adapted programs delivered in other settings (countries, professional sports), and/or to other target groups, and/or with a different public health emphasis (e.g., primary focus on physical activity and sedentary behavior, rather than weight loss).

## 2. Materials and Methods

We describe here the methods used in: (a) ‘retrofitting’ the development of FFIT to the steps described in the PRACTIS guide (Section 2.1); (b) assessing whether scale-up deliveries of FFIT reach the target-group (men aged 35–65 who are at increased risk of future ill-health because of their body size (BMI > 28 kg/m^2^)) and achieve similar outcomes to research deliveries of FFIT (Section 2.2); and (c) mapping scaled-out adaptations of FFIT (delivery to other target groups and/or in other sports settings and/or in other countries) to Aaron et al.’s typology of scaling out (Section 2.3). We do not provide details of methods to assess outcomes in eight ‘scaled-out’ adaptations of FFIT, because they are described in publications referenced in Section 3.3.

### 2.1. ‘Retrofitting’ the Development of FFIT to the PRACTIS Guide

Koorts et al.’s PRACTIS framework [17] had not been published when we first developed FFIT, but we have found it to be a useful framework to describe the steps we took to develop, evaluate and scale-up FFIT from initial pilot deliveries, through the randomized controlled trial (RCT) of FFIT’s effectiveness and cost effectiveness [11], to routine deliveries of FFIT. In doing this, we have followed the ‘retrofitted’ case-studies presented by Koorts et al. (see [17] Additional File 2), to map a ‘retrofitting’ of FFIT’s implementation and scale-up onto the PRACTIS guide’s four steps for successful implementation (see Section 1.2 and Table 1).

### 2.2. Measurement of Pre-Post Outcomes for ‘Routine’ Scaled-Up Deliveries of FFIT

Following a baseline recruitment and measurement session, FFIT comprises 12 weekly group-based 90-min sessions, delivered free of charge to participants by community coaches within professional football clubs (see [11,12,13]). In the pilot [24] and full-scale RCT of FFIT [10,11], baseline measures were undertaken following standardized protocols. As part of their training to deliver FFIT (see Section 3.1.1), football club coaches are trained to measure baseline (week 0) height, weight, waist and blood pressure (BP) and post-program (week 12) weight, waist and blood pressure using very similar standardized protocols to those used by fieldworkers during the FFIT RCT and to give feedback on their objectively measured baseline values As research with FFIT participants demonstrated the importance of this feedback for some men’s motivation to lose weight and modify their lifestyles [25].

To consider whether scaled-up deliveries of FFIT reach the target group, we compare baseline data (means, standard deviation (SD) for weight, waist and blood pressure as indicators of future risk of ill-health) from participants in scaled-up routine deliveries with baseline data from FFIT RCT participants. We also compare the socio-economic characteristics of participants (quintiles of deprivation of area of residence (SIMD)). To consider whether outcomes in scaled-up routine FFIT deliveries are comparable to the FFIT RCT, we compare changes from baseline (week 0) to post-program (12 weeks) in mean values (SD) for key physical measures (weight (kg and %), waist (cm), systolic and diastolic blood pressures (mmHg)).

### 2.3. Methods for ‘Scaled-Out’ Adaptations of FFIT

We have mapped scaled-out adaptations of FFIT (delivery to other target groups and/or in other sports settings and/or in other countries) to Aarons et al.’s typology of scaling-out [14] (as defined in Section 1.2). In drawing on Aarons et al.’s typology, we interpret the population as being the ‘same’ if the target group and key inclusion criteria are the same as tested in the full-scale FFIT RCT (i.e., men, aged 35–65 years, BMI > 28 kg/m^2^). However, we recognize that there can be cultural differences between the target populations described below, meaning a case could be made for regarding the populations from different countries as being different, despite having the same inclusion criteria for age and body composition.

## 3. Results

After summarizing the development and evaluation of FFIT in relation to the PRACTIS guide (Section 3.1), which includes a brief overview of the licensing model developed for scale-up (see Section 3.1.4), we present data on the characteristics and outcome measures for participants in routine FFIT deliveries across almost all of the 42 clubs in the Scottish Professional Football League (SPFL) and in clubs in England following the completion of the FFIT RCT (Section 3.2). We then summarize the process (and where available, the outcomes) of eight ‘scaled-out’ versions of FFIT in relation to Aarons et al.’s typology (Section 3.3).

### 3.1. Retrofitting FFIT to the Steps Outlined in the PRACTIS Guide to Implementation

FFIT is a 12-week group-based, weight management and healthy living program delivered originally to men aged 35–65 years who are overweight (‘population’) in professional football clubs (‘setting’) by trained club coaches to support them in losing weight and maintaining their weight loss long term, through cumulative, sustainable changes in their physical activity, diet (food and alcohol intake, and eating patterns) (‘target outcomes’) (Table 1). From the outset, influenced by the RE-AIM framework [26], we aimed to design a program that could: support behavior change and its maintenance longer-term; be delivered in an existing setting which was thought to appeal to many men; be tested through rigorous evaluation to provide data on feasibility, acceptability, effectiveness and cost-effectiveness; and be relatively inexpensive to deliver.

Table 1 summarizes the mapping of three Phases of FFIT’s development, evaluation and scale-up onto the PRACTIS framework [17], with each Phase summarized in a separate column, mapped against Steps 1 to 4 of the PRACTIS guide. Section 3.1.1, Section 3.1.2, Section 3.1.3 and Section 3.1.4 below describe these phases in detail. In brief, Implementation Phase 1 included program development, pilot deliveries and a pilot feasibility trial and comprised two stages. In Implementation Phase 1, Stage 1 (program development), a multidisciplinary group with expertise in gender and health, physical activity and health, complex interventions, adult learning methods, behavioral change, men’s health and food choice and weight loss, worked iteratively to develop the program. The group drew on evidence on successful lifestyle change programs, their own research on how social constructions of gender relate to men’s health and health behaviors (e.g., [27,28,29,30]) and a weight management approach adopted at a men’s health clinic by one group member [31]. In Implementation Phase 1, Stage 2 (initial testing), we undertook a pilot randomized trial, including a process evaluation, program optimization and mapping of behavior change techniques (BCTs) [32] using Michie et al.’s BCT V1 [33]. Implementation Phase 2 comprised a full scale RCT (and a subsequent follow-up to 3.5 years), including an analysis of cost-effectiveness and a process evaluation. Implementation Phase 3 is the ongoing scale-up of FFIT, i.e., routine FFIT deliveries managed by the Scottish Professional Football League-Trust (SPFL-T), following the development of a licensing model (see Section 3.1.4).

#### 3.1.1. Characterizing the Implementation Setting Parameters for FFIT: Place, People and Process, Provisions and Principles (‘Step 1′ (Koorts, H. et al., 2018)) 

A key underlying principle of FFIT is to use the popularity of professional football amongst many men in the UK as a ‘hook’. Thus, a key component is that the program is delivered within the stadia and facilities of professional football clubs (place) to attract men aged 35–65 years who are overweight. FFIT was designed using adult learning styles which build on existing experience. Coaches adopt an interactive, non-didactic style of delivery, which encourages the use of positive banter with and between men to encourage strong group interactions, mutual support and vicarious learning amongst participants, and between the participants and the club coaches. Each weekly session comprises an ‘educational’ component (~30–45 min), focusing on a topic related to food, physical activity or alcohol. Each session also teaches or rehearses a behavior change technique (e.g., goal-setting, goal review, self-monitoring, relapse prevention). This is followed by group-based physical activity led by the coaches, with each man working to a level appropriate to his current fitness and ability (see [13] for more detail). Throughout, men are encouraged to integrate small, cumulative and sustainable changes to their physical activity and eating practices into their daily lives. The basic program principles and processes have not changed between phases (see Table 1).

People and process: In initial pilot deliveries of FFIT (Phase 1), the program was open to men with a BMI ≥ 27 kg/m^2^, but in the FFIT RCT (Phase 2) and subsequent routine deliveries (Phase 3), the minimum BMI eligibility was increased so men with a BMI ≥ 28 kg/m^2^ are eligible to undertake the program (people) (Table 1). Club coaches (people) are trained to deliver the program to groups of men (an ‘education’ part of each session using the session-by-session coach notes which supplement the participant notes, and group-based physical activity tailored to men’s current level of fitness) (process). During the FFIT feasibility pilot study (Phase 1) and the FFIT RCT (Phase 2), coaches were trained by the FFIT research team. During routine (scaled-up) FFIT deliveries managed by SPFL-T (Phase 3) coaches are trained by SPFL-T.

Provisions: In Phase 1, recruitment leaflets to pilot deliveries displayed club insignia and emphasized the chance to be ‘part of the club’ by joining a program at the club grounds (using phrases like ‘Get off the bench’ and ‘Why don’t you join your club and get into shape?’). In Phase 2 recruitment was extended through greater use of word-of-mouth; local and national newspapers and radio; workplaces; and direct in-stadia approaches to men at pre-season matches by the research team. Recruitment leaflets again stressed the chance to be ‘part of’ the club ((‘Get fit. Shed a few pounds. Become more active at your local Scottish Premier League Club… train with your club coaches for free’). In Phase 3 recruitment is led by clubs and facilitated by SPFL-T mainly using word of mouth, and club and SPFL-T websites and social media.

Coaches are provided with a manual detailing key delivery points each week; participants’ manuals provide accessible information (described positively by men as ‘science but not rocket science’) that mirrors the coach manuals. To facilitate self-monitoring of physical activity in daily life, participants are provided with a pedometer in week 1 and encouraged to work each week to set goals to increase their step count following the graduated Walking for Wellbeing in the West program [34] which encourages individualized, progressive brisk walking goals. In Phases 1 and 2, men were also given a FFIT T-shirt to reinforce the sense of being part of a team.

After engagement with relevant stakeholders about the aims of the program and evaluation, funding for deliveries was provided by Scottish Government and the Football Pools during initial piloting and the full-scale RCT. The Scottish Government provided funding for scaled-up post-trial deliveries in Scotland (see Step 2 and Step 4, Implementation Phase 3).

Principles: Football Fans in Training (FFIT) was developed in 2009/10 (see [13]) with an awareness of prevalent constructions of gender, and was designed to work with, not against, prevailing notions of masculinity [8] to address: the increasing prevalence of male obesity; the lack of weight management programs for men; and the widespread assumptions [35] that men are unwilling to take part in health-promoting interventions. It was designed to be gender-sensitized for men in its context, content and style of delivery [8]. Reviews confirm that programs and innovations which focus on “masculine ideals and gender influence to engage men in increasing their physical activity” [36] (p. 775) have strong potential for promoting men’s health in other areas [36,37,38]. FFIT drew on best evidence from lifestyle change programs in emphasizing the use of behavior change techniques (self-monitoring, implementation intentions, goal setting and review and feedback on behavior) associated with control theory [39], to support cumulative, sustainable changes in daily life. Other elements (e.g., identification of barriers to change, graded tasks) drew on social cognitive theory [40]. Subsequent analysis showed that the approach adopted is compatible with fulfilling the basic needs identified in Self Determination Theory [41,42]: ‘autonomy’ is enhanced through encouraging men to focus on changes which they can integrate into their own lives; ‘relatedness’ is bolstered through the positive ‘team’ spirit [8,10,12,43] and the relationships and support that develop between participants and with the club coaches; and ‘competence’ is enhanced as men learn to apply their increased knowledge about basic nutrition, the benefits of physical activity, and behavior change techniques (see also [44,45]).

FFIT builds on (and has strengthened) existing structures within football to support community programs delivered by club coaches. Post-evaluation, implementation continues through the strong ‘football in the community’ expertise and experiences of SPFL-T (see Table 1; Section 3.1.2 and Section 3.1.4).

#### 3.1.2. Identifying and Engaging Key Stakeholders (‘Step 2′ (Koorts, H. et al., 2018))

While at the time that FFIT was being developed, the potential of professional sports organizations to attract men to public health and health promotion initiatives was recognized (see e.g., [46,47,48]), we were not aware of any studies which had undertaken a rigorous evaluation of a structured and reproducible program delivered in this context. Highly fortuitously, the Scottish Premier League (SPL) had established the Scottish Premier League Trust (SPL-T) in 2009 to seek funding and coordinate community initiatives in the 12 teams in the SPL. From an early stage in developing FFIT (Phase 1), we engaged with the person who represented the newly-formed SPL-T (who was on secondment from Scottish Government) and also met several times with public health representatives from Scottish Government, who agreed (alongside the Football Pools) to fund the costs of the pilot deliveries in 11 clubs, and deliveries of FFIT in the 13 clubs participating in the FFIT RCT (Phase 2). We also met with community coaches from SPL clubs in Phase 1 to outline our ambitions for delivering FFIT through them and their clubs. We responded to some initial skepticism and concerns about coaches not having the ‘right’ experience to deliver a program for overweight and inactive men in mid-life, by maintaining contact and facilitating discussion through the pilot deliveries of FFIT, the coach training and post-delivery workshops with the coaches.

From the outset (Phase 1), we also discussed with coaches, clubs, SPL-T and funders not just the importance of assessing the potential appeal of the program to men, but also of evaluating the effectiveness and cost-effectiveness of FFIT through a pragmatic RCT. This and subsequent engagement, including with the Scottish Professional Football Trust (SPFL-T) (which superseded SPL-T with the reconstruction of the Football League to include all 42 clubs in Scotland in 2013) enabled us to appraise key stakeholders of progress in FFIT’s development and the requirements and conduct of the research. It also allowed us to ensure that the program aligned with Government targets and priorities for the prevention and management of obesity and for men’s health, with constraints and opportunities within clubs, and with priorities for SPL-T/ SPFL-T. Meetings between the core research team (S.W., K.H., C.M.G.) and SPL-T/SPFL-T over the decade since FFIT was first conceived (from first conception to routine delivery and scale-up in Phase 3) proved essential at all stages, particularly in facilitating the pilot and full-scale RCT, and in developing a sustainable model for scale-up in Phase 3 (including a co-developed train the trainer model and materials for long-term deliveries—see Section 3.1.4).

#### 3.1.3. Identifying Contextual Barriers and Facilitators (‘Step 3′ (Koorts, H. et al., 2018))

During Phase 1, the first feasibility pilot deliveries of FFIT took place in 11 of Scotland’s 12 top professional (SPL) football clubs, in autumn (September-December) 2010 and spring (February-April) 2011. At the same time we conducted a two-arm pilot randomized trial, including a mixed methods process evaluation, in two of these clubs [24], with 103 men (aged 35–65, BMI > 27 kg/m^2^) (see [13] for more detail). In the pilot trial, following baseline measurements conducted within the club stadia by a fieldwork team trained to standard protocols, 51 men were individually randomized to take part in FFIT within two weeks, and the remaining 52 were randomized to the waitlist comparison group who undertook FFIT four months later.

The FFIT feasibility deliveries and pilot trial [13,24], and additional research during Phase 1 [8], were crucial in enhancing our initial understanding of potential barriers and facilitators. Participants were very enthusiastic about the context and content of the FFIT program, valuing the coach-led classroom and physical activity components of the weekly sessions, coach expertise and commitment, and the camaraderie and peer-support between participants. The coaches appreciated the session-by-session delivery notes that enabled them to convey key healthy eating and physical activity messages and behavior change techniques in a simple, straightforward manner [13]. Despite some initial skepticism, most coaches were very positive about the impact that they felt they had on participants’ lives and health once they had delivered FFIT. The pedometer and the walking program proved very popular. Pedometers were viewed as a valuable, reliable technological aid that motivated and empowered men in self-monitoring of progress towards self-defined goals. Despite concerns expressed through external peer review of the funding application for the FFIT RCT that the ‘walking’ component may not be acceptable to men, we found “Many men experienced the walking program as a means of regaining fitness, thereby enabling them to also regain valued masculine identities and activities, and a step toward regaining a more acceptable [to them] masculine body” [8] (p. 57), while “bolstering masculine identities through occupancy of a valued masculinized space” (p. 62).

The pilot trial, and associated Phase 1 feasibility research through observations funded by SPL-T in the ‘non-pilot trial clubs’, also confirmed that: recruitment and retention were adequate; research procedures were generally acceptable (with minor changes); and weight loss and improvements in other outcomes were all sufficiently promising to proceed to a full-scale trial [24]. Results were reported back to key stakeholders—the Scottish Government and the Football Pools (as the main funders for the pilot delivery), SPL-T, the coaches and the clubs-in oral presentations, and a plain English summary report produced for wider dissemination.

During Phase 2, we undertook a two-group, pragmatic RCT (primary outcome weight loss at 12 months), with embedded process evaluation and assessment of cost-effectiveness; procedures and results are described in detail elsewhere [11,12]. Funding from Scottish Government and the Football Pools had been obtained for three deliveries of FFIT (August to December 2011, February to April 2012, August to December 2012), which allowed for a trial with a waitlist comparison arm in which eligible men (aged 35–65, objectively-measured BMI > 28 kg/m^2^) randomly allocated to this group post-baseline measurements could be offered the program after 12 month outcome measurements had been completed. Recruitment to the full trial began in summer 2011 as soon as funding and ethical approvals for the evaluation were confirmed.

We recruited sufficient men to fill places in all three deliveries across 13 professional football clubs. Men in the intervention group began FFIT within two weeks of baseline measurement; those allocated to the 12-month waitlist comparison group were guaranteed a place on FFIT post-RCT in August to December 2012; men allocated to the February deliveries 2012 did not participate in the RCT, but enabled additional research on understanding how the program was delivered and received [25,44,49], without contaminating the RCT or over-burdening participants. 747 men participated in the RCT, and follow-up to 12 months was high (89% in intervention group; 95% in waitlist comparison group). At 12 months, the mean between-group difference in weight loss (adjusted for baseline weight and club) was 4.94 kg (95% CI: 3.95 to 5.94) and percentage weight loss was 4.36% (95% CI: 3.64 to 5.08); men in the intervention group showed substantially better improvement at 12 months than the comparison group in: objectively-measured blood pressure, waist circumference and percentage body fat; and self-reported physical activity, diet (scores for fatty foods, sugary foods, and fruit and vegetable intake) and alcohol intake; and self-esteem, positive and negative affect and physical health-related quality of life. The health economic analysis showed that FFIT was highly cost-effective [11]. The program attracted high risk men; 90% were judged to be at ‘very’ or ‘extremely’ high risk of future ill-health on the basis of their baseline age (mean age 47.1 (SD 7.98)), objectively-measured BMI (35.3 (SD 4.91)) and waist circumference (95.7% > 102 cm) [10]. Without any specific targeting, FFIT attracted men from across the socio-economic spectrum (% living in quintiles 1 [most deprived] to 5 [most affluent] were 18%, 18%, 16%, 22% and 25%, respectively).

Qualitative interviews and focus groups, conducted post-program with participants and coaches, and with men in the non-trial deliveries of FFIT, identified cultural and organizational/provider level facilitators: men were motivated to improve their lifestyles and the football club setting was a powerful ‘draw’; men highly valued the setting and style of delivery, affording them privileged access to the club (both symbolically and physically) and being with ‘men like them’ (like-minded with a shared interest in football/the club, and like-bodied (perceived to be similar in body-size and fitness)). They valued the ‘team’ spirit, and the interactive, non-didactic style of delivery (encouraging good-humored ‘banter’ between participants, and between participants and coaches) enabled them to take on the messages of the program and enjoy increasing their physical activity week-by week, but also supported some of them to renegotiate their identities as men in relation to health and health-related behaviors [8,10,12,43,44]. The support men received often extended to family members, particularly men’s wives and partners [49], suggesting the potential for the program to cascade wider benefits for diet and eating practices to others.

Longer-term follow-up, using the same protocols for measurements as for the FFIT RCT, has shown that men in the intervention group sustained many improvements in health and behavioral outcomes 3.5 years from baseline (e.g., mean weight loss 2.90 kg (95% CI: 1.78 to 4.02); 32.2% weighed > 5% less than at baseline, reduction in systolic blood pressure −3.13 mmHg (95% CI: −5.15 to −1.11)). The level of sustained weight loss compares well with other gender-sensitized weight loss programs for men [50]. In addition, men in the RCT waitlist comparison group who took part in FFIT in the post-RCT routine deliveries showed similar sustained positive changes 2.5 years after attending the program [51]. The follow-up study also demonstrated the cost-effectiveness of FFIT in the medium-term (to 3.5 years) and longer-term (modelled over participants’ lifetime) [45].

#### 3.1.4. Assessing and Addressing Barriers (‘Step 4′ (Koorts, H. et al., 2018))

The Phase 1 feasibility and pilot study identified a few minor amendments that were needed to the program and led to the increase of the minimum BMI eligibility criterion from ≥27 kg/m^2^ to ≥28 kg/m^2^. Feedback to key stakeholders (SPL, clubs, coaches, potential participants, Scottish Government) through oral presentations, a lay report and media coverage of the program helped to raise awareness of the program.

The successful outcomes and cost-effectiveness demonstrated in the FFIT trial [10,11,12] (Phase 2) provided impetus for the scale-up of FFIT (Phase 3). Funding for ongoing deliveries in a scaled-up intervention can pose a major barrier to long-term implementation of evidence-based interventions, and we identified a need for a delivery model which could support both sustainment and sustainability of the program (in Hailemariam et al.’s terms [16]). SPFL-T used the results of the RCT and related FFIT research to make a successful case to Scottish Government to fund continuing deliveries of FFIT in subsequent years.

The research team initially made the FFIT materials available in an easy-access license through the University of Glasgow (UoG) but soon realized more was needed to ensure fidelity and quality of future program deliveries. The research team had trained club coaches to deliver FFIT in the pilot RCT and full-scale RCT (Phases 1 and 2); a more sustainable model was required for the long-term scale-up of FFIT. The reorganization of Scottish professional football in 2013 to encompass 42 clubs in the Scottish Professional Football League (SPFL) (noted above) was again highly fortuitous. Under the new SPFL-T, with enthusiastic and committed leadership to enhance community activities across the 42 SPFL clubs, SPFL-T were well-placed to take on the oversight of training and delivery of FFIT across the expanded League long-term. In 2014–2015, the FFIT research team secured UoG funding to co-develop (with a FFIT coach and SPFL-T staff) a two-day program to train new coaches/clubs to deliver FFIT. This ‘Train the Trainers’ package became a key pillar underpinning the move from an easy-access license to a single-license agreement between the UoG (which holds the IP for FFIT) and SPFL-T, which allowed SPFL-T to oversee the scale-up of FFIT in the UK and beyond in a ‘single-license franchise’ model. SPFL-T led a rebranding of the program promotion materials and website at this time (https://spfltrust.org.uk/projects/football-fans-in-training/). The premise underlying the licensing model was to create a support structure that could underpin and quality-assure the scale-up of FFIT, ensuring that coaches from every club delivering FFIT were trained to deliver the core components, with oversight and support to new and existing clubs where needed (‘sustainment’ as defined by Hailemariam et al. [16]), and ongoing audit of outcomes (‘sustainability’ [16]) for the benefit of the funders of ongoing deliveries, SPFL-T and UoG. The licensing model is thus designed to facilitate scaled up delivery with fidelity and to protect against commercialization, in particular by organizations or industries with health damaging potential (e.g., fast food chains, manufacturers of alcohol, SSBs and other ‘discretionary’ snacks and foods), to maximize benefit for public health.

### 3.2. Monitoring Outcomes in the Scale-Up of FFIT

As noted in Section 3.1.2, funding from Scottish Government has allowed FFIT to continue to be rolled out to new participants each season (Phase 3 Implementation), with training, oversight, support and quality assurance provided by SPFL-T as described above. Between 2013 and 2018 (i.e., post-trial), 3320 men took part in 201 FFIT deliveries across 36 SPFL clubs. The core FFIT research team (C.M.G., K.H., S.W.) hold roughly biannual meetings with SPFL-T to review audit (pre-post program) data from routine deliveries and discuss developments. In addition, data from Scotland are summarized and reported back to Scottish Government who have now provided funding over ~10 years for FFIT deliveries through block funding to SPFL-T, who then reimburse clubs for coach time and other resource use.

#### 3.2.1. Baseline Characteristics of Men Taking Part in ‘Routine’ Scaled-Up Deliveries of FFIT

The mean age at baseline of men participating in these ‘routine’ scaled-up deliveries of FFIT was 47.6 years (SD 9.5), based on 2940 (88.6%) for whom age was recorded. Baseline physical measurements on these 2940 men show that routine FFIT deliveries continue to be successful in attracting the original target group (Table 2) (see also [10]): mean weight at baseline for men in routine deliveries of FFIT was 108.9 kg (SD 18.8)(FFIT RCT intervention (I) group 110.3 kg (SD 17.9)); mean BMI was 35.3 kg/m^2^ (SD 5.37) (FFIT RCT-I 35.5 (SD 5.1)); mean systolic BP was 150.02 mmHg (18.94) (FFIT RCT-I 139.4 mmHg (SD 17.6)); and mean diastolic BP was 90.1 mmHg (SD 11.8) (FFIT RCT-I 88.2 mmHg (SD 10.3)).

Data were routinely collected on level of deprivation (based on home postcode) from the 2016/17 season (and sporadically before). Table 3 shows that (for 1438 participants with complete postcode data), routine FFIT deliveries continue to attract men from across the socio-economic spectrum.

#### 3.2.2. Pre-Post Changes in Outcomes for ‘Routine’ Scaled-Up Deliveries of FFIT

Table 2 also shows that routine ‘scaled-up’ deliveries of FFIT are successful in supporting men to lose a clinically significant amount of weight and achieve positive changes in other clinically important measures. Mean weight loss over the 12-week program was −5.04 kg (SD −5.13). These improvements are comparable with changes seen in the intervention group in the FFIT RCT (mean 12-week weight loss −5.80 kg; 95% CI: −6.33 to −5.27). Over the course of the 12-week program, mean percentage weight loss in the routine deliveries was 4.6%, mean reduction in waist circumference was 6.8 cm, and mean reduction in systolic and diastolic blood pressure was 8.0 and 6.0 mmHg, respectively (see Table 2). In feedback to SPFL-T participants have continued to report how FFIT has transformed their life (e.g., FFIT Season 2015–16 Report SPFL-T). The routine deliveries also continue to have wider impact. A UoG survey (conducted in 2018) suggested some long-term social benefits amongst past participants in Scotland; many continue to meet up to play football together (26 teams set up), continue FFIT-style sessions (six clubs), meet socially (seven clubs), and fundraise for local charities (nine clubs).

Through the FFIT single license franchise model, SPFL-T supported scale-up within at least nine clubs in England between 2014 and 2019. These ‘early adopter’ English clubs acquired funding from a range of sources and received training through SPFL-T to deliver FFIT. They include: Southampton FC (www.southamptonfc.com/news/2016-09-30/saints-foundation-saints-fans-in-training) and Swindon Town FC [52] (from 2014/5 season); Charlton Athletic, Middlesborough (www.mfc.co.uk/news/nhs-chief-backs-football-fans-in-training) and Torquay United (torquayunited.com/tucst-football-fans-in-training-to-return-sign-up-now/) (from 2016/7); Leyton Orient (www.leytonorient.com/2015/12/15/orient-announce-launch-of-new-football-fans-in-training-programme/) and Wycombe Wanderers (www.wycombewanderers.co.uk/news/2019/march/ffit-returns...-with-female-course-to-follow/) (from 2017/8); Blackpool (from 2018/9); and Guernsey (www.guernseyfc.com/news/guernsey-fc-launches-initiative-to-get-men-ffit) (from 2019/20). In some cases, a funder has undertaken its own evaluation of the program (see e.g., http://www.publichealth.southampton.gov.uk/images/ffit-southampton-evaluation-final-01-12-2015.pdf). Table 4 presents baseline and outcome data for these clubs.

Following the success in some of these ‘early adopter clubs’, in December 2019 the EPL Trust announced that FFIT will be rolled out more widely in 2020, using the operating name FIT FANS in England and Wales (https://www.efltrust.com/efl-trust-are-helping-10,000-fans-tackle-their-weight/). FIT FANS is supported by substantial National Lottery funding from Sport England and will be delivered from January 2020 at 30 clubs (Aston Villa, Birmingham City, Blackburn Rovers, Blackpool, Bolton Wanderers, Bradford City, Bristol Rovers, Bristol City, Charlton Athletic, Club Doncaster Foundation, Derby County, Leeds United, Leyton Orient, Luton Town, Middlesborough, Notts County, Oldham Athletic, Plymouth Argyle, Portsmouth, Preston North End, Rochdale, Rotherham United, Sheffield Wednesday, Stoke City, Sunderland, West Bromwich Albion, Wigan Athletic, Tranmere Rovers and Walsall) (https://spfltrust.org.uk/efl-trust-agree-deal-to-license-ffit/).

The data presented here on the ‘scale-up’ of FFIT clearly demonstrate the sustainability (evidence of FFIT continuing to deliver its intended benefit over an extended period of time) and sustainment (“creating and supporting the structures and processes that will allow an implemented innovation to be maintained in a system or organization”) [16] (p. 2 of 12) of FFIT. The facilitators to this success are considered in the discussion.

### 3.3. Scale-Out of FFIT

This section describes ‘scaled-out’ adaptations of the FFIT program in other countries and sports settings, or deliveries within the professional football setting to other target groups and/or with different target outcomes. Mostly, with the exception of EuroFIT, which was initiated by the FFIT research team, these adaptations were initiated by research teams elsewhere (after publication of the FFIT RCT results or following dissemination of the findings at national and international scientific meetings), or by other organizations. In some circumstances (e.g., Fussball Fans im Training; Active Fans; Move like a Pro), the research team/organization had already acquired/applied for funding before approaching the FFIT research team; in others (e.g., Aussie-FIT, Hockey FIT pilot trial, RUFIT-NZ pilot and full-scale trials) research teams invited the core Glasgow-based FFIT research team (K.H., S.W., C.M.G.) to collaborate on a research funding bid. In each case, adaptations were considered to ensure that the programs were culturally appropriate, reflecting an increasing awareness of the importance of context in population health interventions [21,22,53].

Table 5 presents key information about each program in terms of: program name/branding and funding source; setting; target group; and degree of adaptation from the original FFIT concept, program and materials in relation to Aarons et al.’s typology of scale-out [14] and whether any form of evaluation has been reported on each program to date. An overview of each scale-out of FFIT (describing the context, the process and extent of adaptation, means of delivery and results of evaluation) is then summarized in Section 3.3.1, Section 3.3.2, Section 3.3.3, Section 3.3.4, Section 3.3.5, Section 3.3.6, Section 3.3.7 and Section 3.3.8 below.

#### 3.3.1. Fussball Fans Im Training—Deliveries to Men through the German Bundesliga (FFIT-G)

Following very minor adaptations of the FFIT program materials including translation into German, recruitment to Fussball Fans im Training (https://www.fussballfansimtraining.de/) (referred to here as FFIT-G) began from December 2016 [54]. Initially three members of the FFIT-G research team from the Institute for Therapy and Health Research (IFT Nord), who had acquired funding from German Cancer Aid to cover deliveries and evaluation of FFIT-G, visited Scotland to meet with members of the core FFIT research team and SPFL-T, leading to a tripartite agreement for the roll-out of FFIT in German Bundesliga teams.

In accordance with the single license franchising model (see Section 3.1.4), SPFL-T delivered initial training in FFIT delivery and measurements to members of the FFIT-G research team and club coaches in Germany. Minor adaptations of FFIT materials for FFIT-G comprised substituting food items that were popular in Germany (in sessions referring to healthier diet) and the conversion of imperial units to German standards (e.g., liters instead of pints). In line with the focus of the funding organization (German Cancer Aid), some additional content was added on the links between obesity and cancer [54].

The target population was overweight and obese men aged 35–65 years; men were eligible to take part if objective measurements at the pre-program baseline session confirmed men had a waist circumference ≥ 100 cm and BMI ≥ 28 kg/m^2^. Men who answered ‘yes’ to any questions on the German Physical Activity Readiness Questionnaire or who had a resting systolic BP ≥ 160 mmHg, and/or diastolic BP ≥ 100 mmHg, were asked to provide a letter from their physician supporting their participation. If they could not provide a letter, they were excluded from the group-based physical activity within the sessions (but participated in the ‘classroom’ element and pedometer-based walking program).

Between December 2016 and July 2018, 934 men registered for 29 deliveries of FFIT-G across 15 Bundesliga clubs. An evaluation of the short-term (12-week) effects of FFIT-G on weight loss and other outcomes using a pragmatic non-randomized trial included 561 of these men, with 477 allocated to the ‘intervention’ group, mostly by filling available places on a first come, first served basis, and 84 to a comparison group (see [54] for more details). Baseline and post-program measurements were conducted by coaches trained to the standard measurement protocols for FFIT, as described above (Section 3.1.4). Positive health outcomes were seen amongst participants post program, and these compared well with post-program measures in the FFIT RCT. For example, in FFIT-G: mean 12-week weight loss was 6.24 kg (95% CI: 5.82 to 6.66); over 50% of participants lost at least 5% of their baseline weight and 15% lost at least 10%; mean decrease in systolic and diastolic blood pressure was 11.11 mmHg (95% CI: 9.08 to 13.14) and 8.46 mmHg (95% CI: 7.42 to 9.50), respectively; and mean reduction in waist circumference was 7.83cm (95% CI: 7.23 to 8.44). Further details (including positive outcomes on other measures) are provided elsewhere [54].

#### 3.3.2. Hockey FIT—Deliveries to Men through Canadian Ice Hockey Clubs

In 2014, a research team based at London University, Ontario obtained funding from the Men’s Health Charity, Movember, to undertake a pilot pragmatic RCT of FFIT delivered in Canadian ice hockey clubs. The Hockey FIT initiative reflects the popularity of ice hockey in Canada; two-thirds of adult Canadians are said to follow ice hockey as a fan, and 80% identify it as “a key part of what it means to be Canadian” [55] (p. 2507). Hockey FIT is adapted directly from FFIT [56] (p. 3) and uses the “love of [ice] hockey”, rather than football (soccer), as the hook. Hockey FIT’s aim is to “encourage middle-aged, overweight and obese men to lose weight, increase physical activity and live a healthier lifestyle” [56] (p. 3). Minor adaptations “to ensure the content followed Canadian guidelines and would resonate with Canadians” [56] (p. 3) included: modifying language to reflect the North American context (e.g., substituting ‘fries’ for ‘chips’, and ‘cups’ for ‘pints’); replacing resources relevant to Canadian rather than British national guidelines (e.g., substituting Canada’s Food Guide and Canadian Physical Activity Guidelines); and replacing football references with hockey references. Two notable differences from FFIT were that in the two clubs participating in the pilot RCT, the program was delivered by “University level students” [57] (rather than club community coaches as in FFIT) and some sessions were held in a local fitness club rather than at the club’s home arena [56]. Components from Health*e*Steps^TM^ were also included (e.g., “lifestyle prescriptions”, and access to a private online social network platform and Health*e*Steps^TM^ smartphone app). Participants were encouraged to engage with these tools post-program during a “forty-week minimally supported phase” (see [55]). However, process evaluation data from the pilot RCT showed that the Health*e*Steps^TM^ smartphone app “was not used by participants due to technical challenges experienced by the men (i.e., difficulties signing in, not tracking steps, crashing)“ and analysis of interviews with participants 12 months after baseline measures showed that “the Hockey FIT social network was used passively with men only accessing the Heath*e*Steps^TM^ network when they received a message/post from their coach or another participant. Men admitted their own and other participants [sic] lack of interaction on the social network limited the potential of the network to support their progress during the minimally-supported phase” [57] (p. 7).

The target audience for Hockey FIT, like FFIT, is men aged 35–65 years, with objectively measured BMI ≥ 28 kg/m^2^ who “meet safety requirements using the Physical Activity Readiness Questionnaire” [56] (p. 5). To facilitate the adaptation of the Hockey FIT materials and design of the pilot trial, members of the Hockey FIT team from London, Ontario, visited the FFIT research team in Glasgow in January 2015 and observed FFIT sessions as they were being delivered at Scottish clubs.

A pilot pragmatic trial of Hockey FIT [56] has been completed, with the primary objective “to examine the feasibility of recruiting and retaining men in Hockey FIT and the acceptability of the research procedures” [55] (p. 2057). Eighty male fans of two clubs from the Ontario Hockey league were recruited, measured and randomized, either to participate in Hockey FIT soon after the baseline measures (intervention group), or after 12-week follow-up measures had been completed (waitlist comparator group); the intervention group was additionally followed to 12 months. Outcome measures closely replicated those used in the FFIT RCT. Men’s characteristics at baseline (mean age 48.7 (SD 9.0), 95% white ethnicity, mean BMI 36.5 kg/m^2^ (SD 6.0), mean weight 112.2 kg (SD 6.0), mean waist 121.4 cm (SD 12.3), mean systolic BP 138.3 mmHg (SD 15.4) and mean diastolic BP 89.2 mmHg (SD 9.6)–see [55]) suggest that Hockey FIT, like FFIT, attracted men at high risk of future ill-health. Retention to the trial was >80% at 12-week measures, and >75% (for the intervention group) at 12 months. Mean weight loss at 12 weeks was 3.58 kg (95% CI: 1.60 to 4.48) greater in the intervention than in the comparator group. Thirty percent of the intervention group lost at least 5% of their baseline body weight at 12 weeks, compared to 3% of the comparator group. On the basis of their process evaluation, the Hockey Fit team identified some adjustments that could be made, including “improving mid-program attendance, coach training, nutrition education, timing, exercise modifications, amount of hockey skills and drills, app usability, the booster session/reunion, and the Hockey FIT social network”. However, they concluded that “these items were minor and would not require significant changes to the program design” [57] (p. 8). A full-scale RCT of Hockey FIT is ongoing (https://clinicaltrials.gov/ct2/show/NCT03636282).

#### 3.3.3. Move Like a Pro—Deliveries to Men through Professional Rugby Clubs in England

In 2015, Premiership Rugby in England also obtained funding from Movember to deliver a weight management program called Move like a Pro (MLAP) in professional English rugby union clubs. As with FFIT-G and Hockey-FIT, MLAP draws closely on FFIT and uses FFIT materials and session format (delivery over 12, weekly 90-min sessions at club stadia by club community coaches). The UoG FFIT research team made minor adaptations to the program to appeal to male supporters of Premiership Rugby clubs, drawing in part on an earlier UoG-led pilot at Sale Sharks rugby club [65]. They also incorporated some components of the EuroFIT program (see Section 3.3.8), including more emphasis on: sedentary behavior (Session 4); sugar in non-alcoholic drinks (Session 5); social support (Session 9); weight loss maintenance including specific relapse prevention tools (Session 10); men recognizing the benefits of the changes they have made (Session 11); and use of social media to facilitate social support outside sessions. The target population was men aged 35–65 years with a BMI ≥ 28 kg/m^2^.

The MLAP program was piloted in five Premiership Rugby clubs between April and July 2016. The UoG FFIT research team adapted the FFIT measurement protocols and tools to support coach-led data collection on participants’ weight, height, waist circumference, blood pressure, physical activity, sedentary behavior, alcohol intake, diet and wellbeing. Coaches from the five pilot clubs were trained in MLAP program delivery and evaluation protocols in a 3-day workshop in January 2016 led by the UoG FFIT research team and an experienced FFIT coach, in conjunction with staff from Premiership Rugby. The UoG team led a feasibility study to examine: recruitment and retention; the impact of the MLAP program on target outcomes; fidelity of delivery; and participants’ and coaches’ responses to, and experiences of, the program. This included baseline, 3-month and 6-month telephone interviews with participants, coaches and community managers from all clubs, as well as observation of selected session deliveries.

The feasibility study showed that Premiership Rugby’s initial recruitment targets proved over-ambitious; clubs achieved 37.2% (n = 186) of their target of 500 men (100 per club). Although over 70% of men completed the program, staffing problems at one club led to high attrition, and some men’s participation was curtailed by injuries sustained during physical activity. Attrition at measurement follow-up was high (59.1% and 26.9% were measured by coaches at 3 and 6 months, respectively); therefore, mean reported changes in outcomes should be treated with caution, as those attending follow-up measurements may be atypical. Men measured at 3 months lost 3.9 kg in weight (95% CI: 3.1 to 4.7). Fidelity of delivery of the key activities of the MLAP club-based, group program declined as sessions progressed. Coaches tended to devote more time than recommended to the physical activity training sessions, at the expense of the classroom activities.

Participants were broadly positive about the program. Many of those interviewed post-program appreciated the clear, simple and gradual approach to changing physical activity and diet. The camaraderie with like-minded men encouraged them to keep attending, and to make positive lifestyle changes. Men also spoke about new friendships with other participants that had continued after the 12 program sessions, and how their involvement with their family had increased as a result of doing MLAP. Coaches said the program delivery materials made the weekly, in-stadia sessions relatively straightforward to deliver, but some felt more ongoing training or mentoring during delivery would have been beneficial. Most were enthusiastic about the opportunity offered by MLAP to widen their community engagement activities to include inactive, overweight middle-aged men.

In 2019, MLAP continues to be delivered independently of the FFIT research team through Premiership Rugby, with National Lottery Sport England funding, which will also be used to deliver the program to women and through workplaces in England https://www.premiershiprugby.com/in-the-community/breakthru/movelikeapro/ (accessed 11.10.19).

#### 3.3.4. FFIT for Women—Deliveries to Women through Professional Football Clubs in Scotland 

As FFIT was rolled out post-RCT in routine deliveries for men across Scotland (see Section 3.2), many clubs reported a demand for the program to be made available for women, partially reflecting a growth in women’s interest in football [59]. In 2014, SPFL-T approached Scottish Government to request that some of that year’s funding for FFIT be used to cover the cost of a small number of FFIT for Women pilot deliveries. Minimal adaptations to the original FFIT program included changes to dietary recommendations (ideal calorie and alcohol limits) to reflect contemporary recommendations for women, and replacing male pronouns with female pronouns. Clubs were asked to ensure that a female coach was present at all sessions alongside another male or female coach trained to deliver FFIT [59]. The target group was women aged 35–65 years with BMI ≥ 28 kg/m^2^.

A UoG-led feasibility study was conducted in five SPFL clubs between April and November 2014. This included before-and-after measures of objectively-measured weight, waist circumference and blood pressure undertaken by fieldworkers trained to the same measurement protocols used in routine FFIT deliveries. Self-report questionnaires were used to investigate changes in physical activity, diet and alcohol consumption, positive and negative affect and self-esteem. Focus group discussions were conducted after the 12-week programs had completed with participants from all five clubs (see [59] for more detail).

All clubs recruited sufficient participants to run the program (range n = 17–27 women). Mean weight loss (2.87 kg, 95% CI: 2.09 to 3.65) and mean decrease in waist circumference (3.84 cm, 95% CI 2.92 to 4.77) were less than observed in men post-program (12-week follow-up) in the FFIT RCT; however, mean decreases in systolic (8.08 mmHg, 95% CI 4.11 to 12.06) and diastolic (5.15 mmHg, 95% CI 2.32 to 7.98) blood pressure were somewhat greater. Women who participated in the focus group discussions said they appreciated what they perceived as the differences between FFIT for Women and other weight loss programs they had previously attended. In particular, women valued the inclusion of physical activity which they experienced as enjoyable and sociable, and the emphasis on making small, cumulative and sustainable lifestyle changes; most, but not all, women enjoyed the pedometer-based walking program within FFIT [59]. The gender of the coach appeared to be less important to them than the skills and relatability of the coach.

Following the success of these pilot deliveries, FFIT is now available as separate men-only and women-only deliveries across many of the SPFL clubs.

#### 3.3.5. Active Fans—Deliveries to Men and Women through European Football Clubs

Active Fans, deliveries of FFIT together with a ‘Healthy Football League’ (SPFL-T, personal communication), has been available through nine European professional football clubs (PSV, Vitesse Betrokken, Bayer 04 Leverjusen, Ferencvarosi TC, Feyenoord Rotterdam, KAA Gent, Valerenga Fotball, Rangers FC and Fulham FC) between 2018 and 2020. The target population is overweight and obese men and women aged 35–65 years with BMI ≥ 28 kg/m^2^. Very minor adaptations to the FFIT program materials were made, including linguistic translation into local languages. To ensure the materials were also culturally appropriate (particularly in the sessions relating to diet) changes were made to ensure the information provided was relevant locally (e.g., by providing examples of foods that were popular in each country).

SPFL-T delivered initial training in program delivery to coaches from the nine clubs in April 2018. This was supplemented by subsequent training in online data capture and reporting (in September 2018). However, following feedback from the first delivery phase, the measurement and reporting process was changed to adhere more closely to FFIT protocols (SPFL-T, personal communication).

More detailed monitoring findings are due to be published in 2020. Initial results suggest that participants lost on average more than 3 kg (https://www.activefans.eu/news/5th-active-fans-project-meeting-in-budapest/).

#### 3.3.6. RUFIT-NZ—Deliveries to Men through Professional Rugby Clubs in New Zealand

In 2015, a team led from New Zealand (NZ) obtained funding from the NZ Health Research Council and University of Otago to adapt and pilot FFIT for delivery as the Rugby Fans in Training NZ (RUFIT-NZ) in professional rugby clubs in Auckland and Dunedin. This reflected the fact that “Rugby (Union and League) is an integral part of NZ culture, the most popular spectator team sport, with high participation rates, particularly in Maori and Pacific peoples” [60] (p. 3 of 14). The NZ team were “cognizant that changes to the program may be needed to align with the rugby environment and cultural needs of men in NZ. [and that] FFIT’s generalizability to different ethnic groups. has yet to be tested” (p. 3 of 14). A formative process was used to develop RUFIT-NZ, including focus groups and interviews with key stakeholders (overweight men, men’s female partners and key healthcare and health promotion agencies in NZ, including Maori and Pacific health care providers). They raised concerns about safety (a need to screen overweight men with co-morbidities) and alignment with existing services and Maori/Pacific cultural values.

The RUFIT-NZ program was delivered to a different target group (men aged 25–65 years, who self-reported not meeting NZ PA guidelines, and had a BMI > 25 kg/m^2^. Slightly different versions of RUFIT-NZ were piloted in clubs in Auckland and Dunedin (see [60], Table 1 and Table 2, for a detailed comparison of both versions with FFIT). The most notable changes were that the Auckland delivery tested out twice-weekly 90 min sessions (one at the weekend and one in the working week), three nutrition sessions were delivered by Pacific Heartbeat (a community nutrition education group) and the session on alcohol was delivered by the club doctor.

A total of 96 participants were recruited into the pilot RCT (n = 49 intervention; n = 47 controls), 46 from Auckland and 50 from Dunedin. Recruitment was completed within a month. A −2.5 kg (95% CI −5.4 to 0.4) mean weight loss at 12 weeks favored the intervention group, who also showed positive improvements in waist circumference, resting heart rate, diastolic blood pressure, cardiovascular fitness and ‘adherence’ to lifestyle behaviors. All men who were followed up reported that they liked the program and would recommend it to other men [60]. As outcomes were promising and the study confirmed the feasibility and acceptability of RUFIT-NZ, the program (with some adaptations) is currently being evaluated in a full-scale RCT. This RCT will provide important evidence on the relative appeal and effectiveness of the program to Maori and Pacific, and thus “have relevance for men in other countries or regions with high rates of obesity including Tonga and other Pacific Islands, Mexico and South America, where sport is an important part of national identity” [60] (p. 12 of 14).

#### 3.3.7. Aussie-FIT—Deliveries to Men through Professional Australian Rules Football Clubs in Australia

In 2017, a research team led from Curtin University obtained funding from the West Australian Health Promotion Foundation, Healthway, to undertake a feasibility study and pilot RCT of Aussie-FIT. The Aussie-FIT program (http://www.aussiefit.org/) was adapted from FFIT for delivery to men through two Australian Football League (AFL) clubs in Perth, Western Australia. Adaptations were thought necessary because of the new sporting and country context, to take account of differences in culture, health care provision and weather [61]. Adjustments were made to include content and design features to suit the context and culture of Australia (e.g., incorporating existing resources from Australian public health campaigns, such as LiveLighter (https://livelighter.com.au/) and providing participants with links to resources available via national campaigns such as Australia’s Alcohol Think Again resource (https://alcoholthinkagain.com.au). The importance of taking preventive measures for protection from skin cancer when being active outside was also stressed. In addition, the Aussie-FIT program was explicitly designed to incorporate principles of psychological theory, most prominently, Self-Determination Theory (SDT) [41,42]. Thus, the program content (e.g., activities, phrasing in written materials) was designed to particularly emphasize opportunities for participants to experience autonomy, competence and relatedness, the three ‘basic psychological needs’ central to SDT. In addition, a ‘need supportive communication style’ was emphasized throughout the coach training, to help coaches to support men’s basic needs, and in turn, promote more autonomous motives for engaging in increased physical activity and healthier eating practices [61] (p. 3). Aussie-FIT sought to support behavior change maintenance, through an increased focus on habit formation, relapse prevention, problem solving and dealing with competing goals from the onset of the program. It also had an increased focus on reducing sedentary time. Instead of providing basic pedometers (as in the FFIT RCT), men were provided with wrist-worn activity monitors that synchronized with a user-based platform which provided continuous data that participants could access via internet-enabled devices, giving them greater access online to information on their activity, in addition to their step count. This partly reflects changing expectations with rapid technological development, and partly the expressed preference by some FFIT participants for more sophisticated self-monitoring devices [44]. The Aussie-FIT program also provided men with practical tips on the use of technology to support (sustained) weight loss, such as online applications and programs (e.g., MyFitness Pal) [61]. Adaptation was also informed by interviews with male AFL fans (n = 9) and AFL coaches (n = 5), and a survey of 151 male AFL fans, 90.5% of whom indicated—after being shown an informational video about FFIT—that this type of approach would appeal to them.

The PI for the Aussie-FIT feasibility and pilot trial discussed the research design and adaptations with the FFIT research team through teleconferences and a visit to Scotland in January 2017 when she was able to observe FFIT being delivered in a football club, and to talk to men and women who had taken part in FFIT/FFIT for Women.

The target population for Aussie-FIT was men aged 35–65 years with an objectively-measured BMI ≥ 28 kg/m^2^ recruited from the fan base of the Freemantle Dockers and West Coast Eagles Australian football clubs in Perth [61]. Coaches were selected and received 16 h of face-to-face training by the Aussie-FIT research team in how to deliver the program, which included opportunities to practice session delivery and receive feedback from the research team and their peers. Coach training also provided rationale for, and application of, need supportive strategies and behavior change strategies and their relevance to eating and physical activity behaviors.

A feasibility and pilot RCT [61] has been completed. 130 men (at baseline, mean age 45.78 (SD 8.01), mean BMI and mean weight 34.99 (SD 5.67) and 111.41 (SD 18.23), respectively) were screened using the Adult Pre-exercise Screening System, measured according to a standardized protocol and randomly allocated to the intervention group or a waitlist comparison group (who were offered a place on Aussie-FIT after the 12-week follow-up measures had been completed). Both groups were re-measured 6 months post-baseline; these measures showed that weight loss in the intervention group was larger than in the 3-month measures. In addition to the outcome measures included in the FFIT RCT, the Aussie-FIT trial included instruments to measure key constructs allied to SDT (e.g., motivation for weight loss, perceived need support in relation to weight loss, need satisfaction), indicators of use and effectiveness of behavior change strategies emphasized through the program (e.g., measures of automaticity, goal conflict and facilitation, action and coping planning) and measures of sleep quality. In addition, sedentary time and physical activity were objectively measured using waist-worn ActiGraph GTX-9 accelerometers [61]. Results from the Aussie-FIT pilot trial are due to be published in early 2020.

#### 3.3.8. EuroFIT—Deliveries to Men through Professional Football Clubs in England, The Netherlands, Norway and Portugal

**Figure 1:**
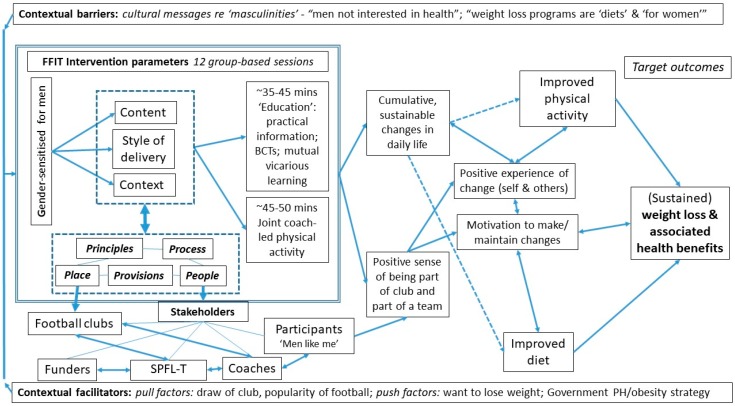
A simplified theory of change for FFIT, showing core components and mechanisms of action in relation to the PRACTIS framework. Footnote: Detail on BCTs, further elucidation of mechanisms of action are available elsewhere [8,10,12,13,25,43,44].

While initiated by the UoG FFIT research team, the EuroFIT research team includes collaborators from the Netherlands, Portugal, Ireland, England and Norway, who brought in new expertise. In 2013, this five-country team obtained funding from the European Commission to adapt, develop and test a program, EuroFIT, designed to support men in making sustainable changes to become more active and reduce sedentary time; for those that wished to lose weight, information to support dietary change was also included [62]. EuroFIT is an evidence- and theory-based 12-week group-based program, building on FFIT [63]. After an extensive period of adaption [63], the EuroFIT program retained key components of FFIT in terms of resources and program inputs, and mechanisms to attract men, and support them in initiating and maintaining changes (see [63] Figure 1, p. 4, for the EuroFIT logic model), while introducing novel elements and resources aligned with change in the primary outcomes (objectively-measured physical activity and sedentary time). To support the change in emphasis from weight loss to physical activity and sedentary time, participants were provided with a bespoke device, the SitFIT^TM^, for self-monitoring both physical activity (step counts) and non-sedentary behavior (upright time), and access to a bespoke app-based game, MatchFIT, which was designed to encourage social support around physical activity between sessions. EuroFIT draws explicitly on motivational theories (Self Determination Theory [41] and Achievement Goal Theory [66]) in addition to sociological theories of masculinity.

An RCT to test the effectiveness and cost-effectiveness of EuroFIT was conducted across 15 football clubs in four European Countries (England, the Netherlands, Norway and Portugal) [62,64]. The target population was men aged 30–65 years with a BMI > 27 kg/m^2^. Club coaches were trained by members of country-based research teams; 1113 men (recruited between September 2015 and February 2016), measured by fieldstaff, also trained by members of the country-based research teams to a standard protocol. As for the FFIT RCT, follow-up to 12 months was high (88% of the intervention group; 92% of the waitlist comparison group), and over 80% in both groups provided objective measures of physical activity and sedentary behavior at 12 months (81% and 85%, respectively). EuroFIT proved feasible to deliver in clubs in all four countries, and participants were positive in their evaluations of the program. EuroFIT was effective in increasing physical activity at 12-month follow-up (estimated difference in increase in objectively measured step count 678 steps/day (97.5% CI: 309 to 1048) in favor of the intervention group), even though men recruited to the trial already had quite high baseline step counts (mean 8372 steps/day); however, there was no reduction in sedentary time in either group at 12 months, despite some reduction in sedentary time post-program [62]. Analysis of extensive process evaluation data [63] and of underlying mechanisms [64] across the four countries is ongoing. A lower mean difference in weight loss at 12-month follow-up (−2.4kg, 95% CI −3.1 to −1.7) between EuroFIT intervention group participants versus the comparison group [62] in comparison with the difference in weight loss between men in the two arms of the FFIT RCT [11] is likely to reflect differences in the target behaviors and primary outcomes of the two trials, and hence, for example, the later introduction in the EuroFIT program of information relating to healthy eating and dietary choice) [62].

## 4. Discussion

Milat et al. have noted the “growing body of literature describing frameworks for scaling health interventions” but highlight that, despite this: “the lag between evidence generation and implementation represent[s] a considerable impediment to population-wide health improvement as it denies or delays community access to effective services. Even where there is evidence of the efficacy or effectiveness of public health interventions, *there has been much less attention paid to the mechanisms*” [23] (p. 1 of 11, emphasis added).

Similarly, Koorts et al. note the lack of evidence for the “successful institutionalization” of physical activity and similar public health interventions in real-world settings and the gap in “‘*how to*’ enact strategies to successfully translate research into practice” [17] (p. 2 of 11, emphasis in original) and Peters et al. suggest that more practice-based evidence is needed to address the research-to-practice gap rather than research conducted in highly controlled circumstances [21]. In this paper, we describe one public health intervention, a weight management and healthy living intervention (Football Fans in Training, FFIT), gender-sensitized to appeal to and engage men, which has been implemented widely within a relatively short space of time both within its original setting and, through different degrees of adaptation, beyond. In exploring this example of relatively rapid implementation, we have described not just where FFIT is now delivered and embedded, but have attempted to draw out some of mechanisms and some practical ‘how to’ principles in relation to two implementation frameworks: Koorts et al.’s recently published PRACTIS guide and Aarons et al.’s distinction between ‘scaling-up’ and ‘scaling-out’. We discuss each in turn.

### 4.1. Scale-Up

We have shown through clearly describing the program’s 5 ‘Ps’ (Place, People, Process for delivery, Provisions needed for delivery and Principles) (Koorts et al.’s Step 1), and with clear identification of facilitators and barriers and strategies to overcome them, and the involvement of key stakeholders throughout three phases of implementation that we have been able to scale-up the FFIT program from feasibility work and a pilot randomized trial (Phase 1), through a full scale RCT and long-term follow-up (Phase 2), to routine deliveries in a scaled-up model (Phase 3). This is summarized in a Theory of Change model drawing out key components of FFIT in Figure 1.

The data presented here also clearly demonstrate the sustainability (“the extent to which an evidence-based intervention can deliver its intended benefit over an extended period of time”) and sustainment (“creating and supporting the structures and processes that will allow an implemented implementation to be maintained in a system or organization”) [16] (p. 2 of 12)) of the FFIT program. A key step was establishing a sustainable model of delivery, through a single license franchising model, which has provided a vehicle for rapid integration into routine practice in Scotland and beyond and builds on the partnership between the core FFIT research team and SPFL-T. The premise underlying the licensing model was to create a support structure that could underpin and quality-assure the scale-up of FFIT. The support structure ensured that coaches in every club delivering FFIT were trained to deliver the core components (with oversight and support where needed) and to conduct baseline and post-program measures using standard protocols, and that outcomes were audited to provide some assurance about quality of delivery. The scale-up baseline and post-program outcomes show that FFIT continues to attract its intended target group, and supports weight loss and other health benefits that are comparable with results delivered in research conditions. Key factors in this successful scale-up relate to the good fit of the key components of the FFIT model with dominant constructions of masculinity [8,12,13]. In particular, the physical, social and symbolic context of the professional football setting has proved a powerful ‘hook’ to engage men in the program; the content has proved relevant, useful and accessible in supporting men to make sustained changes to their weight, waist size, blood pressure, wellbeing, physical activity and eating patterns; and the style of delivery has suited and actively engaged men, creating an atmosphere in which they can relax, learn and take part in some group-based physical activity in an enjoyable manner that fits with their identities as men.

The scale-up of FFIT also demonstrates alignment with most of the ‘scalability considerations’ that Milat et al. [23,67] suggested should be addressed when scaling up health promotion interventions. These are: “contextual factors”; “effectiveness, reach and adoption”; “costs”; “workforce, technical and organizational resources required”; “intervention delivery”; and “appropriate evaluation approaches” (see [67] p. 290, Table 2 for “sub-themes” for each of these “themes”). We briefly consider FFIT in relation to Milat et al.’s themes and sub-themes in turn below.

With respect to contextual factors, the “interaction of the intervention with individual, community, cultural, political, workforce and organizational contexts” ([67], p. 290) was considered from the outset. First in relation to the wider political context, the benefits shown for FFIT are relevant to several priority areas for public health (obesity, inactivity, cancer, heart disease, hypertension, mental health and wellbeing) within Scotland and other parts of the UK. Key underlying principles of FFIT were to work with not against prevailing cultures of masculinity [8,10,13] and to use the popularity of sport, and in particular in the UK football, as a ‘draw’. In doing this, from the outset we approached what was then the newly-formed Scottish Premier League Trust (which became the SPFL-T in 2013), in the hope that such an overarching structure may have the potential to deliver and oversee the program long-term. Milat et al. noted that:

“identifying the most efficient and sustainable workforce and organizational infrastructure at the trial stage may not always be possible; but if it is done, it may accelerate the adoption of effective interventions more widely into policy and practice”.

[67] (p. 294)

This is probably a crucial factor in the rapid scale-up of FFIT. The fact that many football clubs in the UK have a community department also provides an ideal and sustainable workforce at club level to deliver the program. These community coaches are valued by participants for their skills in both providing non-didactic direction in ‘classroom’ parts of the sessions and leading the group-based physical activity; their banter with participants, weaving in background knowledge of the clubs and players, is crucial in making participants feel ‘part of the club’ and in fostering team spirit. The single-license franchise model with SPFL-T has provided a successful organizational infrastructure for wider delivery. The ‘Train-the-Trainers’ model ensures that delivering coaches and clubs have the required technical expertise and training to deliver FFIT, and the collection of pre- and post-program data allows for evaluation and performance monitoring. The single-license franchise model has enabled us to address the “workforce, technical and organizational resources required” and the integration of quality control and performance monitoring systems (see [67], p. 290) has enabled an “appropriate evaluation approach” during scale-up, as recommended by Milat et al. ([67], Table 2, p. 290).

Another key factor in FFIT’s scale-up has been building robust evidence on effectiveness and cost-effectiveness of the program, including that beneficial outcomes are sustained not just 12 months after joining the program [11] but also to 3.5 years from baseline [51]. Understanding the costs of an intervention and so the value for money is particularly important when public health bodies have many priorities to meet, and the demonstrated benefits and evidence of short-medium and long-term cost-effectiveness are persuasive for funding bodies, particularly those who have an evidence-based approach to decision-making on spend. In terms of reach and adoption, we have shown here that FFIT continues to reach and engage men at high risk of future ill-health, on the basis of their age, BMI, waist circumference [10] and blood pressure, from all socio-economic backgrounds. Its continuation throughout clubs in Scotland under the auspices of SPFL-T, with funding from the Scottish Government, and the recent announcement that FFIT will be rolled out as FIT FANS across 30 clubs in the English Football League from January 2020, with ambitions to tackle obesity levels in over 10,000 fans across the UK (https://www.efltrust.com/efl-trust-are-helping-10000-fans-tackle-their-weight/) evidences its adoption by different settings and organizations. This widespread uptake of “intervention delivery” and testimonies on participating club websites and social media, also evidences the continuing acceptability to participants and to other stakeholders, as first shown in our more detailed research in the first two phases of implementation of FFIT [8,10,12,13,43,44].

### 4.2. Scale-Out

This paper also demonstrates that the FFIT program can readily be translated for delivery in a range of different sports and countries and to different populations, with greater or lesser degrees of adaptation—that is that it can be scaled-out. Crucial to this process, as for the scale-up of the model, has been the widespread popularity and cultural embeddedness of professional sport, together with robust evidence on effectiveness and cost-effectiveness of the program. The place of sport in many men’s sense of identity, and indeed sometimes people’s national identity, as a ‘hook’ has transferred well to other settings and countries. This suggests great potential for other sports and, in more rural or remote areas, for the use of local sporting clubs. Here we have documented scale-out of FFIT into football and other professional sporting contexts in Australia, Belgium, Canada, England, Germany, Hungary, the Netherlands, New Zealand, Norway and Portugal to date, and there is increasing interest in programs for women which adopt a more holistic and sustainable approach to weight management than many commercially available ‘diets’ which are targeted at women, as evidenced by the popularity of FFIT amongst some women in more recent years.

Movsisyan et al. noted that: “decisions on when, to what extent, and how to adapt interventions are not straightforward, particularly when conceptualising intervention effects as contingent upon contextual interactions in complex systems” [68]. Aarons et al. argued that, with all types of scale-out, an intervention must: (i) retain its core elements; (ii) retain the underlying mechanism of action linking these core elements to health outcomes and (iii) have “sufficient organizational or system support to deliver the intervention as intended to sufficient numbers of the target population” [14] (p. 4). Figure 1 depicts a simplified theory of change for FFIT, highlighting its core components and underlying mechanisms of action. We would argue that the scale-outs of FFIT which are described here satisfy the first two conditions, but it is too early in their development and initial deliveries to be sure that they can each be supported longer term by sufficient organizational or system support, or deliver the same long-term results. Some Type 1 scale-outs (e.g., FFIT-G [54]), have been able to demonstrate good outcomes (as evidence of successful translation) through less labor intensive and expensive evaluation by “borrowing strength”(in Aarons et al.’s terms [14]) from evidence of effectiveness from the earlier FFIT RCT. Those which are examples of Type 3 scale-out (EuroFIT and RUFIT-NZ) have been [62] or are currently being (RUFIT-NZ) evaluated in a full-scale RCT.

Peters et al. have noted that “Context plays a central role in implementation research. Context can include the social, cultural, economic, political, legal, and physical environment, as well as the institutional setting, comprising various stakeholders and their interactions, and the demographic and epidemiological conditions” [21] (p. 1 of 7). The various translations of FFIT described here show that relatively small adjustments to the program have been needed for it to be successful in attracting men in other countries, although the model has yet to be tested in, for example, lower and middle income countries where the passion for sport is deep-rooted but many aspects of context will differ radically from the UK. Most of the scale-outs to date, as described here, have involved minor adaptations to the original program, involving ‘surface’ rather that ‘deep structure’ modifications (i.e., “harmonizing intervention materials (e.g., handbooks as part of manualized interventions) to observable characteristics of the target population, such as using culturally appropriate messages, language, and product brands to improve outward appeal, acceptance and face validity” (see [68]).

In addition to direct adaptations of the program, FFIT has provided the impetus for other programs for men based in sport settings. For example, a 12-week program, HAT TRICK (http://hattrick.ok.ubc.ca/), has been developed at the University of British Columbia (UBC) and successfully piloted in Canadian ice hockey clubs in British Colombia. While this program draws to some degree on the experience of FFIT, the informational resources, tailored messaging and weekly PA and dietary tracking logs are based principally on the UBC team’s prior research on gender-sensitized interventions for men (e.g., [69,70]). Its name reflects the program’s tripartite goals (to increase physical activity, encourage healthy eating and strengthen social connections amongst men) and the design of the program materials “aligns with participant identities as ice hockey fans and men” [71] (p. 2159). The pilot feasibility trial of HAT TRICK is reported elsewhere [72].

Evidence on what works for men’s health is accumulating rapidly, and programs that focus on “masculine ideals and gender influence to engage men in increasing their physical activity” have been shown to have strong potential for promoting men’s health [36,37,38]. Engaging men through ‘doing’ something [38], attracting them to engaging and trusted environments (places in which they are comfortable with people they perceive has having much in common (‘people like me’) and providing them with skills and accessible information (‘science but not rocket science’) that they can apply in day-to-day life going forward appear to be crucial components in the scale-up, scale-out and continuing success of FFIT. We would argue that the gender-sensitization in context, content and style of delivery have been central to the success of FFIT (taking account of the gender in the ‘5Ps’ (Place, People, Process, Provisions, Principles) in Koorts et al.’s terms [17]). However, the importance of stakeholder engagement throughout, allowing all relevant parties to highlight facilitators and raise and resolve barriers (Koorts et al.’s Steps 2–4) cannot be overstated. Further research is needed to better understand the growing appeal of the program’s approach to women.

## 5. Conclusions

This body of research, and research conducted in Australia [50,73,74,75,76,77,78,79,80] and Canada (e.g., [36,69,70,72], demonstrates that, with attention to cultural constructions of masculinity in relation to health and health behavior, public health interventions can be gender-sensitized so that they both appeal to men and support them in sustainable lifestyle changes, which may cascade to other family members and to wider society. The successful scale-up and scale-out of FFIT, as documented here, profoundly contest the view that men ‘won’t’ take part in such programs and present an ongoing challenge for the public health community to find the right ‘hooks’ to engage men. The evidence provided on the rapid scale-up and scale-out of an effective and cost-effective program demonstrates the success in the wider implementation of Football Fans in Training in practice. Professional sports settings have proved a powerful ‘hook’ for engaging men, but are a finite resource, and thus, should be used as a setting for public health interventions that have a strong evidence base. We have also shown that the FFIT approach and setting is appealing to some women. As the scale-up and scale-out of FFIT continues to evolve, the efforts of SPFL-T and the English Football League (EFL) Trust to secure Sport England funding to deliver FFIT (as FIT FANS) to up to 10,000 men and women in England from January 2020 is evidence of its relevance and appeal as one strand in the complex public health response to growing levels of obesity and physical inactivity in many countries of the world.

## Figures and Tables

**Table 1:** FFIT development, evaluation and scale-up mapped onto the ‘5Ps’ (Place, People & Process, Provisions, Principles) and Steps 1–4 of the PRACTIS guide.

	Implementation Phase 1: Program Development, Pilot Deliveries & Pilot Feasibility Trial	Implementation Phase 2: FFIT RCT and Long-Term Follow-Up	Implementation Phase 3: Routine FFIT Deliveries
*Target outcome*	*Weight loss, physical activity (PA), diet*	*As for Phase 1+ behavior change maintenance*	*As for Phase 2*
*Population*	*Men (35–65 yr, BMI ≥27 kg/m^2^)*	*Men (35–65 yr, BMI ≥28 kg/m^2^)*	*As for Phase 2*
*Setting*	*Professional football clubs in Scotland*	*As for Phase 1*	*As for Phases 1 & 2*
**Step 1: Characterize implementation setting parameters**	**Place**:	**Place**:	**Place**:
	In-stadia facilities of 11 Scottish Premier League (SPL) clubs.	In-stadia facilities of 12 SPL clubs + club most recently relegated from SPL.	In-stadia facilities of 36/42 Scottish Premier Football League (SPFL) clubs & professional football clubs in England.
	**People** & **Process**:	**People** & **Process**:	**People**& **Process**:
	12-week intervention targeting men who are overweight/obese. Aimed to recruit~30 men for program delivery facilitated by two club coaches (participant: coach ratio ~15:1).	As for Phase 1 + ‘Light touch’ maintenance (emails, invite to reunion).	As for Phase 1 (i.e., ‘Light touch’ maintenance not continued).
	Community coaches from the clubs trained by research team over 2 days (a few clubs engaged external male health trainers to work alongside community coaches).	As for Phase 1 (except coaches from several clubs had experience of delivering FFIT in pilot phase).	Community coaches from the clubs trained by SPFL-T over ~2 days.
	Support with any issues during 12-week delivery from research team.	As for Phase 1.	Support with any issues during 12-week delivery from SPFL-T.
**Step 1 (contd.)**	Recruitment via club with support of research team: club website, leaflet mailings, word of mouth (including emails), newspapers (local and national), other (e.g., adverts in local venues, match day advertising.	Recruitment via club with support of research team: word-of-mouth; local, national & social media; workplaces; direct approaches in-stadia to men at pre-season matches.	Recruitment via: club resources; SPFL-T website & social media; word-of-mouth; local, national & social media; NHS referral.
	**Provisions**:	**Provisions**:	**Provisions**:
	**Recruitment** flyers; **Participant & coach manuals** detailing key delivery points week-by-week, with space for men to record progress against goals; **Coach training** delivered by research team; Research fieldwork team trained to standardized protocols to **measure outcomes** at baseline, 12 weeks, 6 months and 12 months in two clubs participating in pilot RCT.	As for Phase 1.	SPFL-T website & staff to support FFIT **recruitment** and deliveries; Social media & recruitment materials (including participant endorsements); **Participant & coach manuals** (as for Phase 1); **Coach training** delivered by SPFL-T (using ‘train the trainers’ model and materials) coaches trained to **measure outcomes** (pre-post) to standardized protocols (refresher training for delivery and measures every 3 years).
	**Pedometer** to self-monitor daily step count.	As for Phase 1.	**Pedometer**/own device to self-monitor daily step count.
	**Funding** from Scottish Government & Football Pools to reimburse clubs/coaches for their time/resource use.	As for Phase 1.	**Funding** from Scottish Government to reimburse SPFL-T (for training, oversight, audit) & clubs/coaches for their time/resource use Oversight & audit of outcomes by SPFL-T (with input from research team biannually to review outcomes & updates).
**Step 1 (contd.)**	**Principles**:	**Principles**:	**Principles**:
	**Intervention**—Gender-sensitized, working with not against dominant cultural constructions of masculinity in relation to health; targets small, cumulative, sustainable changes in daily life to support weight loss, increased physical activity & healthy eating. Develops participant skills in toolkit of BCTs, e.g., goal setting, self-monitoring, problem solving, identifying social support, encouraging participants to select what works for them. Designed to be delivered using club facilities and coaches at relatively low cost.	**Intervention**—As for Phase 1.	**Intervention**—As for Phase 1.
	**Implementation**—Builds on and develops existing structures within clubs; congruence with aims and aspirations of newly-established Scottish Premier League Trust (SPL-T); congruence with public health priorities to address rising obesity, poor diet and physical inactivity.	**Implementation**—As for Phase 1, building on skills and experience acquired during pilot deliveries of FFIT.	**Implementation**—As for Phase 1 + uses infra-structure & experience of FFIT & other ‘football in the community’ initiatives in SPFL-T as an overarching organizational structure, supporting community coaches within clubs to deliver health-promoting programs to adults.
**Step 2: Identify and engage key stakeholders**	Newly established SPL-T; Coaches and clubs within SPL.	As for Phase 1.	SPL-T became SPFL-T in 2013; Coaches & clubs within the SPFL & football clubs elsewhere in the UK.
**Step 2 (contd.)**	Scottish Government and Football Pools as funders for the deliveries of FFIT.		Scottish Government as funder for ongoing, routine deliveries of FFIT in Scotland.
	Men in mid-life who are overweight.		Men in mid-life who are overweight.
	Advisory group (including academic, funder and SPL representation).		Oversight group from UoG (core FFIT team).
**Step 3: Identify contextual barriers and facilitators**	**Cultural level facilitators** (identified through process evaluation in pilot trial): (i) ‘push factors’ (large pool of men who are overweight/physically inactive and want to make changes but not attending other weight management services); (ii) ‘pull factors’ (the ‘draw’ of the club and the popularity of football/ professional sport, and positive association with men’s interests).	**Cultural level facilitators** (identified through process evaluation in RCT): As for Phase 1.	**Cultural level facilitators**: As for Phase 1.
	**Organizational and provider level facilitators**: (i) most clubs have a more or less developed community wing of club, with community coaches; (ii) availability of research team to deliver coach training; (iii) funding to provide materials (FFIT-branded t-shirts, FFIT/club-branded manuals, pedometers) and cover	**Organizational and provider level facilitators**: (i) most clubs have developed or developing community wing, with community coaches with some experience of delivering FFIT in pilot stages; (ii) availability of research team to deliver coach training; (iii) developing infrastructure within SPL-T to support clubs delivering FFIT in recruitment; (iv)	**Organizational and provider level facilitators**: (i) availability of gold-standard evidence on effectiveness and cost-effectiveness; (ii) well-developed and expanded infrastructure within SPFL-T to train club coaches to deliver FFIT; (iii) most clubs have an established community wing of club, with community coaches with experience of delivering FFIT; (iv)
**Step 3 (contd.)**	coach time and room bookings for measurement sessions.	support from FFIT research team in recruitment at pre-season ‘friendly’ games to ensure full recruitment to all available deliveries; (v) skills of club coaches in engaging groups of participants and delivering group-based activities, with increased experience of and confidence in delivering to men in mid-life.	developing reputation of FFIT (‘word-of-mouth’) and club-based/SPFL-T social media to facilitate recruitment to new deliveries; (v) well-established expertise within SPFL-T; (vi) ‘licensing model’ to provide structure for training, oversight, support and administration by SPFL-T; (vii) train the trainers materials, protocols and experience provided by SPFL-T; (viii) periodic meetings between SPFL-T and UoG to review progress, developments, quality assurance and outcomes.
	**Cultural barriers**: popular messages reinforcing men’s lack of interest in health, and weight loss programs as ‘diets’ and ‘for women’.	As for Phase 1.	As for Phase 1 (potentially decreasing with time).
**Step 3 (contd.)**	**Organizational and provider level barriers**: (i) community coaches’ lack of experience in working with target group (overweight men in mid-life); (ii) skepticism about whether FFIT can be delivered in the context and can support intended target outcomes; (iii) lack of experience in clubs of undertaking research, particularly using a randomized design; (iv) competing demands on coach time and club facilities.	**Organizational and provider level barriers**: (i) competing demands on coach time and club facilities.	**Organizational and provider level barriers**: (i) lack of fully-developed model for routine scale-up in initial post-trial deliveries, prior to the development of licensing model.
**Step 4: Address & assess barriers**	Process evaluation: Interviews and discussions with stakeholders (SPL-T, club coaches) provided feedback to guide development of intervention processes and materials. Interviews conducted with coaches, participants, and drop-outs from the program.	Process evaluation: Extensive process evaluation as part of FFIT RCT to identify remaining barriers to implementation, and barriers to change for participating men. Documented recruitment methods, interviews/ focus groups about coach and participant experiences post-program.	Formative evaluation: Additional impact accelerator grant to develop a ‘train the trainer’ model and materials. Work by SPFL-T to ‘rebrand’ FFIT.
	Observations of pilot deliveries across the clubs. Feedback from advisory group.	Observations of program delivery.	Regular quality assurance involves attending some session deliveries.
**Step 4 (contd.)**	**Strategies to address barriers**: Training and support for delivery for club coaches, provided by research team. Development of publicity materials in anticipation of positive results from the pilot (‘video’ diary of sessions by BBC journalists, BBC Radio documentary). 5-a-side tournament between participating clubs as awareness raising/media coverage in anticipation of recruiting to full RCT.	**Strategies to address barriers**: Intensive effort to recruit sufficient men for trial within short period between funding decision and trial start.	**Strategies to address barriers:** Development of licensing model, including mechanisms to audit pre-post outcomes in routine (post-RCT) deliveries of FFIT, allowing regular feedback to funder on numbers participating, characteristics of participants and outcomes on key measures. Increasing size and capacity of SPFL-T staff to train clubs to deliver with fidelity, oversee and evaluate process and outcomes and for wider communication and advocacy for increased uptake in new clubs. Development of online data collection tool from 2019 to improve quality of data and enhance reporting
	**Process/outcome evaluation**: Led to minor changes to eligibility criteria (BMI at least 28) and program as a result of formative evaluation.	**Process/outcome evaluation:** Extended learning about mechanisms and key components for successful delivery.	**Process/outcome evaluation**: Periodic (biannual) meetings between SPFL-T and UoG to review audit data on routine deliveries.

**Table 2:** Physical measures at start (baseline) and end (post-program) of routine deliveries of FFIT in Scotland (mean [SD]) (n = 2940/3320 men who had no missing data on age).

	n	At Baseline (Pre-Program)	n	12 Weeks (Post-Program)	Change
Weight (kg)	2932	108.9 (18.8)	2534	103.7 (18.0)	−5.0 (5.1)
Weight (%)		-		-	−4.6 (3.9)
BMI (kg/m^2^)	2606	35.3 (5.4)	2333	33.3 (5.4)	−1.7 (1.6)
Waist (cm)	2855	115.0 (17.2)	2468	108.0 (17.3)	−6.8 (7.4)
Systolic BP (mmHg)	2672	150.0 (18.9)	1803	142.1 (17.2)	−8.0 (14.6)
Diastolic BP (mmHg)	2671	90.1 (11.8)	1803	84.0 (9.9)	−6.0 (10.4)

**Table 3:** Socioeconomic characteristics of participants in routine deliveries of FFIT in Scotland.

SIMD ^+^ Quintile	Frequency	Percent
1 (most deprived)	339	23.6
2	305	21.2
3	286	19.9
4	251	17.5
5 (least deprived)	257	17.9
Total	1438	

^+^ Scottish Index of Multiple Deprivation derived from postcode of residence.

**Table 4:** Physical measures at start (baseline) and end (post-program) of routine deliveries of FFIT in nine clubs in England (mean [standard deviation SD]).

	n	At Baseline (Pre-Program)	n	12 Weeks (Post-Program)	Change
Weight (kg)	308	110.4 (16.7)	262	104.0 (16.4)	−6.3 (4.6)
Weight (%)		-		-	−5.7 (4.0)
Waist (cm)	307	115.0 (18.8)	2468	107.0 (18.7)	−7.9 (5.4)
Systolic BP (mmHg)	290	144.5 (16.8)	228	137.5 (15.8)	−7.7 (12.0)
Diastolic BP (mmHg)	290	88.4 (10.1)	228	83.9 (9.1)	−4.6 (7.8)

**Table 5:** Adaptation of FFIT in other professional sports settings, countries or target groups.

Name of Adapted Program (Funding)	Country; Sport Setting; Target Group	Degree of Adaptation; Using Aarons et al.’s Typology	Evaluation	Publications
Fussball Fans im Training (German Cancer Aid)	Germany; Football; Men, aged 35–65, BMI ≥ 28 kg/m^2^, waist circumference ≥ 100 cm	Very minor; Type 1 scale-out	Pragmatic non-randomized trial, waitlist comparison group	Pietsch, Weisser [54]
Hockey-FIT (Movember [pilot], CI HR [RCT])	Canada; Ice hockey; Men, aged 35–65, BMI ≥ 28 kg/m^2^	Very minor; Type 1 scale-out	Pilot pragmatic RCT completed [55]; full RCT ongoing	Gill, Blunt [56]; Petrella, Gill [55] Blunt, Gill [57]
Move like a Pro (Movember [pilot])	England; Premiership Rugby; Men aged 35–65, BMI ≥ 28 kg/m^2^	Very minor; Type 1 scale-out	Small scale feasibility study completed	Gray et al. [58]
FFIT for women (Scottish Government)	Scotland; Football; Women aged 35–65, BMI ≥ 28 kg/m^2^	Minor; Type 2 scale-out	Feasibility study [59]; analysis of further data ongoing	Bunn et al. [59]
Active Fans (Erasmus + Sport)	Belgium, England, Germany, Hungary, Netherlands, Football; Men and women, aged 35–65; BMI ≥ 28 kg/m^2^	Minor; Type 1 and 2 scale-out	Pre-post measures, as for FFIT scale-up	-
RU-FIT NZ (Health Research Council, NZ	New Zealand; Rugby; Men, aged 25–65, BMI ≥ 25 kg/m^2^	Moderate; Type 3 scale-out	Pilot RCT completed [60]; full RCT ongoing	Maddison et al. [60]
Aussie FIT (Healthway)	Australia; Aussie-Rules football; Men aged 35–65; BMI ≥ 28 kg/m^2^	Moderate; Type 1 scale-out	Feasibility and pilot RCT completed [61]	Quested et al. [61]
Euro-FIT (European Commission)	England, NL, Norway, Portugal; Football; Men aged 30–65, BMI ≥ 27 kg/m^2^	Substantial; Type 3 scale-out	Full RCT completed [62]	Wyke, Bunn [62], van de Glind, Bunn [63]; van Nassau, van der Ploeg [64]
